# LEOPARD syndrome: clinical dilemmas in differential diagnosis of RASopathies

**DOI:** 10.1186/1471-2350-15-44

**Published:** 2014-04-26

**Authors:** Claudia Santoro, Giuseppe Pacileo, Giuseppe Limongelli, Saverio Scianguetta, Teresa Giugliano, Giulio Piluso, Fulvio Della Ragione, Mario Cirillo, Giuseppe Mirone, Silverio Perrotta

**Affiliations:** 1 Dipartimento della Donna, del Bambino e di Chirurgia Generale e Specialistica, Second University of Naples, Via Luigi De Crecchio, 4, Naples 80138, Italy; 2 Division of Cardiology, Second University of Naples, Monaldi Hospital, Naples, Italy; 3 Department of Biochemistry, Biophysics and General Pathology, Second University of Naples, Naples, Italy; 4 Dipartimento di Scienze Mediche, Chirurgiche, Neurologiche, Metaboliche e dell’ Invecchiamento, Second University of Naples, Naples, Italy; 5 Department of Pediatric Neurosurgery, Santobono Children’s Hospital, Naples, Italy

**Keywords:** LEOPARD syndrome, Neurofibromatosis type 1, RASopathy, PTPN11

## Abstract

**Background:**

Diagnosis within RASopathies still represents a challenge. Nevertheless, many efforts have been made by clinicians to identify specific clinical features which might help in differentiating one disorder from another. Here, we describe a child initially diagnosed with Neurofibromatosis-Noonan syndrome. The follow-up of the proband, the clinical evaluation of his father together with a gene-by-gene testing approach led us to the proper diagnosis.

**Case presentation:**

We report a 8-year-old male with multiple café-au-lait macules, several lentigines and dysmorphic features that suggest Noonan syndrome initially diagnosed with Neurofibromatosis-Noonan syndrome. However, after a few years of clinical and ophthalmological follow-up, the absence of typical features of Neurofibromatosis type 1 and the lack of *NF1* mutation led us to reconsider the original diagnosis. A new examination of the patient and his similarly affected father, who was initially referred as healthy, led us to suspect LEOPARD syndrome, The diagnosis was then confirmed by the occurrence in both patients of a heterozygous mutation c.1403 C > T, p.(Thr468Met), of *PTPN11*. Subsequently, the proband was also found to have type-1 Arnold-Chiari malformation in association with syringomyelia.

**Conclusion:**

Our experience suggests that differential clinical diagnosis among RASopathies remains ambiguous and raises doubts on the current diagnostic clinical criteria. In some cases, genetic tests represent the only conclusive proof for a correct diagnosis and, consequently, for establishing individual prognosis and providing adequate follow-up. Thus, molecular testing represents an essential tool in differential diagnosis of RASophaties. This view is further strengthened by the increasing accessibility of new sequencing techniques.

Finally, to our knowledge, the described case represents the third report of the occurrence of Arnold Chiari malformation and the second description of syringomyelia with LEOPARD syndrome.

## Background

Noonan syndrome (NS, OMIM 163950), Neurofibromatosis type 1 (NF1, OMIM 162200), LEOPARD syndrome (LS, OMIM 151100), and Neurofibromatosis type 1-like syndrome (NFLS, OMIM 611431) belong to the group known as RASopathies [1]. They are characterized by overlapping phenotypic features; each one also comprises key features that can help to differentiate one from another. NS is relatively common; it affects approximately 1/1000 to 1/2500 newborns. NS is characterized by postnatal reduced growth, distinctive facial dysmorphisms, cardiac defects, and variable cognitive impairment in association with other features, including ectodermal and skeletal defects, cryptorchidism, lymphatic dysplasia, bleeding tendency, and increased risk of developing malignancies during childhood [2-4]. Diagnoses are made by clinical examination according to a comprehensive scoring system [5]. NS is characterized by extreme clinical and genetic variability [6].

Clinical diagnosis of NF1 is based on the presence of two or more of the following signs: six or more café-au-lait macules (CALMs); two or more neurofibromas of any type or one plexiform neurofibroma; axillary or inguinal freckling; optic glioma; two or more Lisch nodules of the iris; a distinctive osseous lesion; and/or a first-degree relative diagnosed with NF1 according to the preceding criteria. Some of these features, such as spots and plexiform neurofibromas, are usually already present at birth or during the first years of life [7]. On the other hand, Lisch nodules and cutaneous neurofibromas tend to become evident after 6 years of age [8]. Neurofibromatosis-Noonan syndrome (NFNS) represents a specific entity in which features of both NS and NF1 can be recognized [9].

The acronym LEOPARD stands for the cardinal features of LS: lentigines, electrocardiographic (ECG) conduction abnormalities, ocular hypertelorism, pulmonary stenosis, abnormal genitalia, retardation of growth, and sensorineural deafness [10]. LS patients show a phenotype that strongly overlaps with NS, although they are characterized by a typical dispersed pattern of multiple lentigines and CALMs, which are less frequently observed in NS [11]. A diagnosis of LS is made on the observation of key features such as lentigines, hearing loss and hypertrophic cardiomyopathy (HCM) [5]. The same as for NF1, these clinical signs of LS appear to be age related. Thus, NF1, NS, and LS phenotypes may be indistinguishable in young children and infants.

NFLS is a recently identified RASopathy characterized by a mild NF1 phenotype with pigmentary changes, macrocephaly, learning difficulties, and a tendency to develop lipomas in adulthood. Lisch nodules and neurofibromas are typically absent [12].

RASopathies always result from germline mutations of genes that encode protein components of the Ras/MAPK (mitogen-activated protein kinase) pathway [13]. Mutations in *NF1* are detected in approximately 95% of patients fulfilling the NIH clinical criteria [14]. NS can be caused by mutations in several genes, including *PTPN11*, *KRAS*, *SOS1*, *RAF1*, *NRAS*, *BRAF*, *SHOC2*, *CBL and RIT1*; *PTPN11* changes account for roughly 50% of cases [6,15]. *PTPN11*, *BRAF*, and *RAF1* mutations are also mutated in LS and are responsible for approximately 95% of cases, with mutations in *PTPN11* alone occurring in about 85% of cases [16]. De Luca et al. provided strong evidence of a major role for *NF1* mutations in NFNS [9], while *SPRED1* mutations have been identified in patients with NFLS [12].

Herein, we describe a child who initially fulfilled the diagnostic criteria for NF1, but who later did not develop typical features of NF1, such as Lisch nodules and neurofibromas. After negative results for *NF1* and *SPRED1* mutations, clinical examination of the patient and his family history led us to suspect LS, which was subsequently confirmed by *PTPN11* testing. Intriguingly, this led us to a diagnosis of LS also in the proband’s father.

## Case presentation

**Figure 1:**
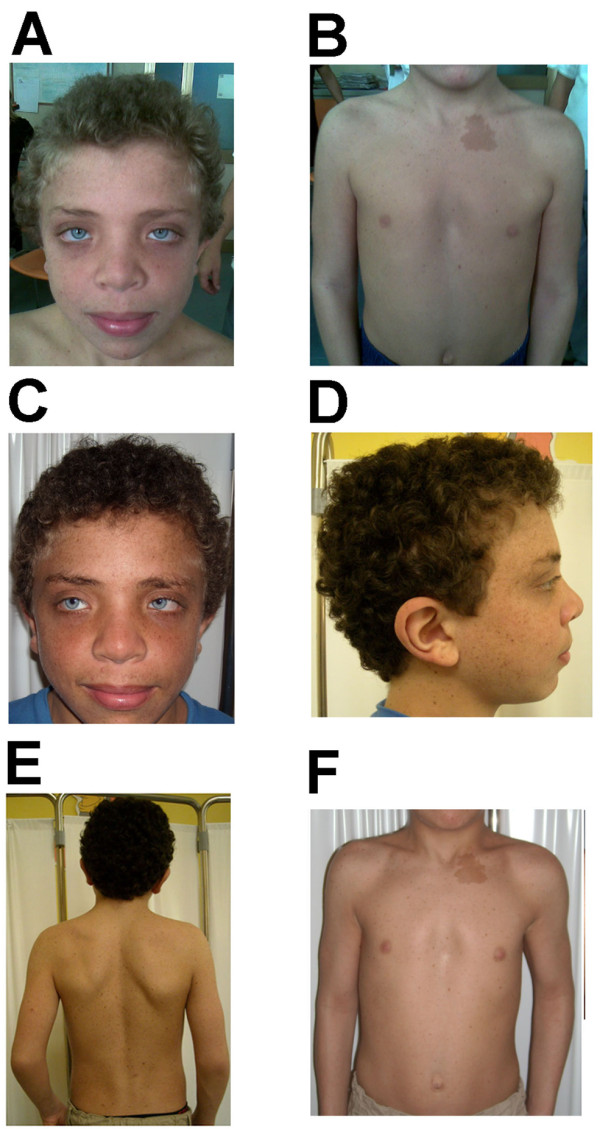
**Photographs of the proband at 8 (A, B) and 12 (C-F) years of age. (A)** At the age of 8 the proband patient had blue eyes, epicanthus, down-slanting palpebral fissures, hypertelorism, coarse facial features, facial lentigines, and curly blond hair. **(B)** Pterigium colli, pectus excavatum, hypertelorism, lentigines, and a large CALM were noted. At the age of 12 years, the proband showed **(C)** persistence of the facial dysmorphism previously noted; **(D)** low-set and posteriorly rotated thick, large ears with lobe creases; and **(E, F)** low posterior hair line and CALMs and an increased number, more widespread pattern of distribution, and deeper color intensity of lentigines.

The proband was the first male child of non-consanguineous healthy parents. He was spontaneously delivered after an uncomplicated pregnancy that was the result of *in-vitro* fertilization. Birth weight was 3.7 Kg (50th centile), and length was 50 cm (50th centile). At 8 years of age, he was referred to our center because of multiple CALMs (six, ≥5 mm). Examination also revealed several light-brown lentigines on his face, neck, and thorax. Dysmorphic features included hypertelorism; downslanting palpebral fissures; epicanthus; coarse facial features; and large, thick, low-set ears. Low posterior hairline, blond curly hair, pterygium colli (Figure 1A), cubitus valgus, chest deformity (Figure 1B), and umbilical herniation were also noted. The boy weighed 25 kg (50th centile), with a height of 126 cm (50th centile) and a cephalic perimeter of 55 cm (95th centile). No genital or hearing anomalies were detected. Based on these features, a clinical diagnosis of NFNS was suspected and the child underwent a multidisciplinary follow-up, as advised for NF1 [17], including periodic visual assessment [18].

A cranial MRI was performed because of headache; no unidentified bright objects (UBOs) were detected. Lisch nodules and neurofibromas were absent. ECG revealed no abnormalities at the age of 9 years. Because mutations in *NF1* are known to be the major molecular event underlying NFNS, molecular genetic analysis of *NF1* was performed. However, no *NF1* mutations were found. Because of the lack of typical NF1 features (i.e., Lisch nodules and neurofibromas) and elements that suggested a particular RASopathy, a molecular analysis of *SPRED1* was launched when he was 11 years of age. No mutations were detected. These negative molecular results led us to reevaluate our initial clinical diagnosis. Thus, we asked to review the child along with both of his parents.

**Figure 2:**
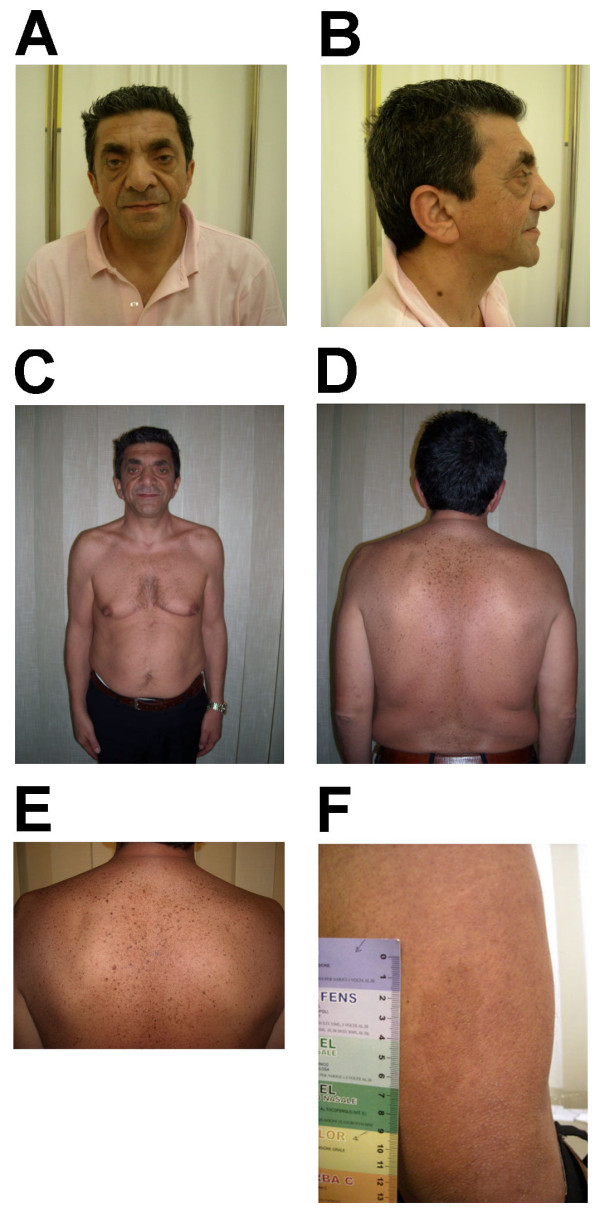
**Photographs of the proband’s father at 50 years of age.** The proband’s father had **(A)** coarse facial features, hypertelorism, and ptosis; **(B)** large, thick, low-set ears with lobe creases; **(C)** pectus excavatum and hypertelia; **(D)** low posterior hair line; **(D, E)** lentigines; and **(F)** a large CALM on the left leg **(F)**.

The proband’s father, who was aged 50 years, came to us first. He had always been referred as healthy, but a history of benign arrhythmia, as well as post-natal onset of growth retardation and peptic ulcer emerged from an in-depth clinical anamnesis. The father had dysmorphic features. Obvious hypertelorism; ptosis and coarse facial features; large, thick ears with creased lobes; low posterior hairline; chest deformity; multiple sparse, dark, small spots and common nevi; a few CALMs; and a large macule on the leg (Figure 2) were detected. No genital problems were present. His height was 166 cm (5th centile), and his head circumference was 60 cm (>95th centile). His father and one of his brothers were reported to share similar facial dysmorphisms without other referred medical problems, such as deafness or cardiac problems. The proband’s uncle had also undergone surgery because of unilateral cryptorchidism.

Upon examination at the age of 12 years, the proband’s CALMs, dysmorphic features (Figure 1C, D) and macrocephaly were confirmed and stable. However, the lentigines had acquired a pattern of distribution more suggestive of LS and their color was darker than when previously observed (Figure 1D–F). Thus, we began to suspect LS.

**Figure 3:**
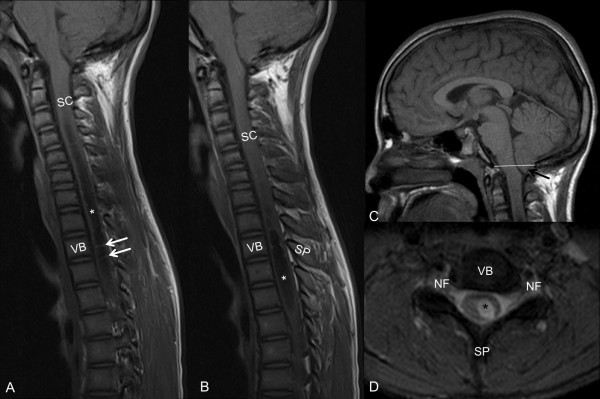
**Magnetic resonance image of the proband.** Sagittal SE T1-weighted cervical MRI **(A, B)** shows cerebrospinal fluid (CSF)-filled cavity (*) in the middle of the central cervical and thoracic spinal cord (SC), with multiple caudal septations. These non-neoplastic septated (white arrows) paracentral fluid containing cavitations are referred to as hydrosyringomyelia. Sagittal SE T1-weighted brain MRI **(C)** with the basion-opisthion line (BOL) shown in white. Note crowded foramen magnum and the low-lying pointed tonsil with distance from BOL >5 mm (black arrow), consistent with Chiari malformation 1. Axial MERGE T2*-weighted cervical MRI **(D)** shows a well-demarcated CSF cavity (*) in the middle central and left paracentral SC. VB, vertebral body; NF, neural foramina; SP, spinous process.

An echocardiography revealed mild dilatation of the ascending aorta and hypertrophic cardiomyopathy in the father and thickening of the intraventricular septum with an ejection fraction (EF) of 70% and mitral valve prolapse in the proband. Mixed mild deafness was demonstrated in the father, but no auditory problems were detected in the proband. Molecular genetic testing of *PTPN11* was performed, and a previously described pathological heterozygous mutation c.1403 C > T, p.(*Thr468Met*), was detected in both the proband and his father. The proband recently underwent an MRI scan of the spine because of thermal and pain hypoestesia of the upper left arm. A syrinx in the cervical spine, extending into the thoracic spine, and a type-1 Arnold-Chiari malformation were detected (Figure 3).

All patients gave informed written consent on entering the study, which had been approved by the Second University of Naples, Ethics Committee, in accordance with the Declaration of Helsinki.

## Discussion

A new class of genetic disorders, the “RASopathies”, has recently emerged. To date, this nosologic group comprises NS, LS, Hereditary gingival fibromatosis type 1, Capillary Malformation-AV malformation syndrome, NF1, NFLS, Costello syndrome, Cardio-facio-cutaneous syndrome (CFC), and Autoimmune Lymphoproliferative syndrome (ALPS) [13]. Collectively, these conditions represent relatively common monogenic disorders. Although each one exhibits typical phenotypic features, they share many clinical signs, including craniofacial anomalies; congenital heart defects; short stature; varying degrees of neurocognitive impairment; cutaneous and musculoskeletal abnormalities and predisposition to malignancies. Thus, differential diagnosis can represent a clinical dilemma, particularly among NF1, NS, LS, and NFLS.

Several case reports of patients with borderline phenotypes that illustrate this clinical overlap have been published. For example, Wu et al. [19] reported a *de novo NF1* mutation in a woman with a prior diagnosis of LS. Moreover, Carcavilla et al. [20] reported three children who filled NF1 clinical criteria but were diagnosed with LS and carried the *PTPN11* mutation p.(*Thr468Met*), the same mutation detected in our patients. Carcavilla et al. [20] suggested that a distinguishing feature for LS might be the diffuse pattern of lentigines, even if they seem to appear later in life than CALMs; this is in accord with our personal observations. The patients described in this previous report had cardiomyopathy but lacked other features typical of LS, such as deafness or genital abnormalities.

Our patients similarly lacked these types of clinical signs. We noted a strong dysmorphic feature overlap between our young patient and the one described by Nystrom et al. [21]. The child described by Nystrom et al. [21], who had a clinical diagnosis of NS because of pulmonic stenosis and NS facial dysmorphisms, was discovered to carry the *BRAF* mutation *p.(Lys499Glu).* The authors proposed a gene-based classification of RASopathies and suggested that NS and CFC are allelic disorders of *BRAF*. A *BRAF* analysis was performed for our proband, and no mutation was found. Taking into account the dysmorphic overlap between our proband (clinically diagnosed with LS and carrying a *PTNP11* mutation) and Nystrom’s patient (diagnosed with NS due to *BRAF* mutation), we enforce the opinion that a straightforward genotype–phenotype relationship is not always present among RASopathies.

In the last 10 years, we did experience with other similar cases. A 3-year-old boy was addressed to us because of CALMs (n° 5) in suspicion of NF1. He showed mild Noonan like dymorphisms, pulmonary stenosis and was a sporadic case. We performed *PTPN11* analysis as first genetic test that led us to NS diagnosis. The other patient was a 18 months-old female with few CALMs and lentigines who initially received diagnosis of NF1. Intriguingly, her parents were healthy. During the following two years she received diagnosis of HCM and developmental delay. Sparse and slow growing hair and Noonan dysmorphisms were also present. The *PTPN11* analysis detected a mutation compatible with LS. All these observations point to the *PTPN11* gene, instead of *NF1*, as the first gene to be ruled out if the differential diagnosis of NFNS/NFLS/LS. On the contrary, a male with CALMs, joint laxity, frontal bossing, macrocephaly, pulmonary stenosis and no lentigines at the age of 5, suspected to be affected by NS, received diagnosis of NF1 after the molecular analysis (firstly performed because of the number of CALMs = 6).

We also report diagnosis of NFLS in 4 patients (3 familiar cases, and one sporadic), and their affected relatives, who previously received diagnosis of NF1. Due to the lacking of NF1 typical stigmata, *NF1* analysis was performed but any mutation was detected. Nevertheless the clinical diagnosis of NF1 was confirmed. After a long follow-up (4–10 years), and after the detection of *SPRED1* mutations as the genetic cause of NFLS, the diagnosis was changed.

The recent description of NFLS produced new questions, particularly about the limits of the diagnostic criteria for NF1 and the clinical homogeneity of this condition. The current clinical diagnostic criteria for RASopathies seem to be inadequate, especially when the patient is observed during childhood, when some age-dependent manifestations are not yet present. Patients who are young and who appear to lack a familial history of RASopathy, need to be clinically reviewed and other specific consultations (e.g., ophthalmological, dermatological, cardiological and audiometric) should be performed in order to reach a correct diagnosis. Unfortunately, these additional evaluations may be associated with burdensome health care costs. Muram et al. [22] reported a cost saving approach by *SPRED1* analysis in patients with a probable NF1 diagnosis. The genetic testing was cost saving between the ages of 9 and 15 years in individuals with multiple CALMs with or without freckling compared to the no-testing approach with routine follow-up. Furthermore, Lepri et al. [23] reported a next generation analysis (integrated by Sanger sequencing of the remaining not-covered regions) resulting 6 time less expensive than protocols entirely based on Sanger sequencing in 10 patients affected by RASopathies.

Our experience with the proband and his father, taken together with the known broad intrafamilial variability of the phenotype in RASopathies, is a reminder of how clinical examinations of parents and other first-degree relatives (although referred to as healthy) can be useful in designing suitable diagnostic approaches to children with suspected NF1/NS/LS/NFLS. In our case report, the clinical evaluation of the father would have helped to orientate the stratified molecular diagnoses, priorizing *PTPN11* analysis. The clinical examination of both parents should be considered in the genetic counselling of couples subjected to the *in vitro* fertilization.

Finally, to our knowledge, this is the third report of the occurrence of Arnold Chiari malformation [24,25] and the second one of syringomyelia with LS [24]. Instead NS is already known to have a high incidence of spinal cord malformations [26] and there are some evidences that this is also true for NF1 [27]. This confirms that LS and NS belong to a clinical spectrum together with NFNS [28].

## Conclusions

In summary, our observations and those described in previously published reports support the indication of molecular testing of *NF1* and other RASopathies genes in cases in which the diagnosis of RASopathy is uncertain or in which the phenotype is borderline. Genome sequencing might represent the right approach for screening RASopathies. This would allow physicians to conduct proper follow-up and genetic counseling, thus reducing the medical and economic costs associated with incorrect diagnoses of RASopathies.

## Consent

Written informed consent was obtained from the patient’s parents and from the father for publication of this case report and any accompanying images. A copy of the written consents are available for review by the Editor of this journal.

## Abbreviations

NS: Noonan syndrome; NF1: Neurofibromatosis type 1; LS: LEOPARD syndrome; NFLS: Neurofibromatosis type 1-like syndrome; NFNS: Neurofibromatosis-Noonan syndrome; CALMs: Café-au-lait macules; ECG: Electrocardiographic; MAPK: Mitogen-activated protein kinase; UBOs: Unidentified bright objects; EF: Ejection fraction; CFC: Cardio-facio-cutaneous syndrome; ALPS: Autoimmune lymphoproliferative syndrome.

## Competing interests

The authors declare that they have no competing interests.

## Authors' contributions

CS conceived of the study, and participated in the design of the study and drafted the manuscript. GP has been involved in the clinical and multidisciplinary diagnosis of the patients. GL has been involved in the clinical and cardiological diagnosis of the patient. SS carried out the molecular genetic studies. TG has been involved in molecular analysis and in acquisition of data. GP has been involved in revising the report critically. FDR has been involved in drafting the manuscript. MC has provided MRI images and has made substantial contributions to conception of the manuscript. GM has been involved in the multidisciplinary approach to diagnosis. SP participated in the study design and coordination and drafted the manuscript. All authors read and approved the final manuscript.

## Pre-publication history

The pre-publication history for this paper can be accessed here:

http://www.biomedcentral.com/1471-2350/15/44/prepub
